# Controlling in-gap end states by linking nonmagnetic atoms and artificially-constructed spin chains on superconductors

**DOI:** 10.1038/s41467-020-18540-3

**Published:** 2020-09-18

**Authors:** Lucas Schneider, Sascha Brinker, Manuel Steinbrecher, Jan Hermenau, Thore Posske, Manuel dos Santos Dias, Samir Lounis, Roland Wiesendanger, Jens Wiebe

**Affiliations:** 1 Department of Physics, Universität Hamburg, D-20355 Hamburg, Germany; 2 Peter Grünberg Institut and Institute for Advanced Simulation, Forschungszentrum Jülich & JARA, D-52425 Jülich, Germany; 3 Department of Physics, RWTH Aachen University, 52056 Aachen, Germany; 4 I. Institute for Theoretical Physics, Universität Hamburg, D-20355 Hamburg, Germany; 5 Present Address: Institute for Molecules and Materials (IMM), Radboud University, Nijmegen, The Netherlands

**Keywords:** Electronic properties and materials, Magnetic properties and materials, Superconducting properties and materials, Topological defects

## Abstract

Chains of magnetic atoms with either strong spin-orbit coupling or spiral magnetic order which are proximity-coupled to superconducting substrates can host topologically non-trivial Majorana bound states. The experimental signature of these states consists of spectral weight at the Fermi energy which is spatially localized near the ends of the chain. However, topologically trivial Yu-Shiba-Rusinov in-gap states localized near the ends of the chain can lead to similar spectra. Here, we explore a protocol to disentangle these contributions by artificially augmenting a candidate Majorana spin chain with orbitally-compatible nonmagnetic atoms. Combining scanning tunneling spectroscopy with ab-initio and tight-binding calculations, we realize a sharp spatial transition between the proximity-coupled spiral magnetic order and the non-magnetic superconducting wire termination, with persistent zero-energy spectral weight localized at either end of the magnetic spiral. Our findings open a new path towards the control of the spatial position of in-gap end states, trivial or Majorana, via different chain terminations, and the realization of designer Majorana chain networks for demonstrating topological quantum computation.

## Abstract (web summary)

Topologically trivial and Majorana bound states can show spectral weight near the ends of a chain of magnetic atoms on a superconductor. Here, the authors disentangle the two contributions by augmenting a spin chain with orbitally-compatible nonmagnetic atoms, where a persistent zero-energy spectral weight at the transition between the two parts is observed.

## Introduction

Candidate Majorana platforms based on magnetic chains^1–7^ have been experimentally realized by self-assembled growth of chains of Fe^8–12^ or Co atoms^13^ on superconducting Pb(110), and by the controlled atom-by-atom assembly of Fe chains on superconducting Re(0001) using the tip of a scanning tunneling microscope (STM)^14^. For both substrates, scanning tunneling spectroscopy (STS) supplied experimental evidence for topological superconductivity in Fe chains via the observation of zero-energy spectral weight localized at the ends of such chains, which was interpreted as the signature of a Majorana bound state^15,16^. For the Pb system^12^, it was shown theoretically that the Majorana bound state can have a strong spectral weight in the superconducting substrate close to the chain’s end, which was experimentally supported by the detection of zero-energy spectral weight localized in Pb overlayers covering the Fe chains close to their ends. However, for all investigated systems so far, the experiments suffer from a concurrence of the expected location of the Majorana bound state with the location of the termination of the chain. This termination is intrinsically different from the inner part of the chain, as the end atoms have a different coordination^17^. Thereby, these atoms tend to have a different bonding length and, thus, a different hybridization with the substrate as compared to those in the interior of the chain. Moreover, the spin-related properties at the ends of magnetic chains can differ drastically from their interior^18–20^. All this could lead to a localization of topologically trivial Yu–Shiba–Rusinov (YSR) states at the chain’s ends^9,13,17,21,22^ and also accidentally close to the Fermi energy, and, therefore, hinder the identification of the spectral weight stemming from the sought-after Majorana bound states. A solution to these issues would be the termination of the magnetic chain by a nonmagnetic superconducting wire made from a material with a similar orbital structure: such a termination ensures a smooth electronic continuation of the magnetic chain, which we expect to have a stronger impact on the properties of the YSR states than on those of the Majorana bound state, due to the robustness inherent to the topological character of the latter. In order to investigate the possibilities to realize such terminations, we study artificial chains of three different species from the *3d* transition-metal series (Mn, Fe, and Co), as well as hybrid chains of Co and Fe atoms, assembled by STM-tip induced manipulation on superconducting Re(0001).

## Results

### Construction of the chains via atom manipulation

**Fig. 1:**
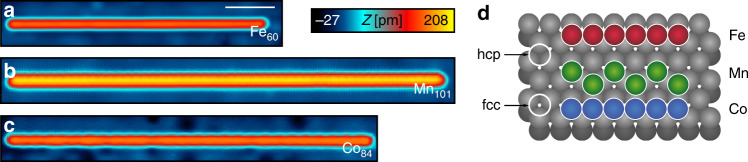
Geometric structures of artificial 3d transition-metal chains built on a Re(0001) surface. **a** Constant-current scanning tunneling microscope (STM) image of a linear chain of 60 hcp Fe atoms. The white scale bar corresponds to 3 nm. **b** Constant-current STM image of a zig-zag chain consisting of 101 Mn atoms, with adsorption sites alternating between the fcc and hcp hollow sites on the Re substrate. **c** Constant-current STM image of a linear chain of 84 hcp Co atoms. All data (**a**–**c**) are recorded with *V* = 6 mV, *I* = 0.2 nA. **d** Sketches of the atomic positions in (**a**–**c**).

By depositing the three transition-metal elements onto the cold Re(0001) substrate and subsequent STM-tip induced manipulation (see “Methods”), we place single Fe and Co atoms on two different adsorption sites: the hollow site that continues the hexagonal close-packed (hcp) stacking of the substrate, and the hollow site that corresponds to the stacking of a face-centered cubic (fcc) crystal. For single Mn atoms, only the fcc site is accessible^23^. Due to increasing Kondo coupling with increasing *d*-state filling and a transition from out-of-plane to easy-plane magnetic anisotropy, the energy of the YSR states, which the five species induce in the energy gap of the superconducting substrate, varies systematically from Mn^fcc^ over Fe^fcc^, Fe^hcp^, and Co^fcc^ to Co^hcp^: the YSR states of Mn^fcc^ are located close to the superconducting substrate’s gap edge, the ones of Fe^hcp^ close to the center of the gap, and the ones of Co^fcc^ again at the gap edge. Interestingly, single Co^hcp^ atoms are found to have fully quenched magnetic moments and thus do not induce any in-gap state^23^. Moreover, artificial chains of Fe^hcp^ atoms on neighboring sites (Fig. 1a) form spin spirals and reveal strong indications for topological superconductivity by zero-energy spectral weight localized at the ends of chains that are longer than ten atoms^14^. Motivated by these previous results, we explore the possibilities to build chains of the other two elements, Mn and Co, on neighboring sites using STM-tip induced manipulation (Fig. 1b, c).

We find that it is possible to manipulate straight chains of Co atoms on neighboring hcp sites (Fig. 1c) and zig-zag-shaped chains of Mn atoms adsorbed on neighboring sites alternating between fcc and hcp (see Fig. 1b, d, Supplementary Note 1, and Supplementary Fig. 1). The chains are up to more than 100 atoms long limited by the widths of the terraces of the substrate, residual substrate defects, and the number of available single atoms in the surrounding of the building area. However, it was impossible to build any other chain of closed-packed atoms of one of these three elements, e.g., straight chains of Mn atoms on neighboring fcc or hcp sites, or straight chains of Co atoms on neighboring fcc sites. This is most probably a result of an energetically unfavorable bond length in such configurations.

### In-gap electronic structure of homoatomic chains

**Fig. 2:**
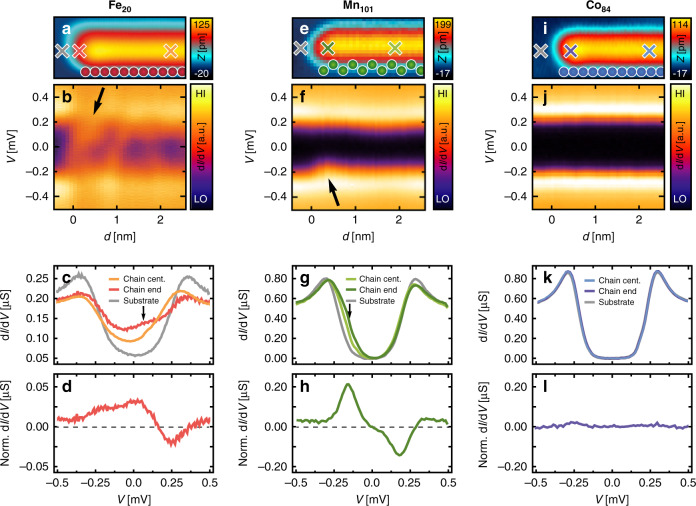
Low-energy electronic structure at the terminations of the Fe, Mn, and Co chains. **a** Constant-current scanning tunneling microscope (STM) image of the end of a Fe_20_ chain. **b** Differential tunneling conductance along the Fe_20_ chain aligned with the topography shown in (**a**). **c** d*I*/d*V* spectra of the Fe_20_ chain and substrate taken at the positions marked by crosses in (**a**) (*V*_stab_ = 1 mV, *I*_stab_ = 0.2 nA, and *V*_mod_ = 20 µV). **d** d*I*/d*V* spectrum on the chain’s end from (**c**) normalized by subtraction of a spectrum averaged along the chain’s interior. **e** Constant-current STM image of the end of a Mn_101_ chain. **f** Differential tunneling conductance along the Mn_101_ chain. **g** d*I*/d*V* spectra of the Mn_101_ chain and substrate taken at the positions marked by crosses in (**e**) (*V*_stab_ = 1 mV, *I*_stab_ = 0.5 nA, and *V*_mod_ = 40 µV). **h** d*I*/d*V* spectrum on the chain’s end from (**g**) normalized by subtraction of a spectrum averaged along the chain’s interior. **i** Constant-current STM image of the end of a Co_84_ chain. **j** Differential tunneling conductance along the Co_84_ chain. **k** d*I*/d*V* spectra of the Co_84_ chain and substrate taken at the positions marked by crosses in (**i**) (*V*_stab_ = 1 mV, *I*_stab_ = 0.5 nA, and *V*_mod_ = 40 µV). **l** d*I*/d*V* spectrum on the chain’s end from (**k**) normalized by subtraction of a spectrum averaged along the chain’s interior. All data (**a**, **e**, **i**) are recorded with *V* = 6 mV, *I* = 0.2 nA. Arrows in (**b**, **c**, **f**, **g**): see text.

Next, we study the low-energy electronic properties of three manipulated chains, as shown in Fig. 1, in the energetic region of the energy gap 2Δ = 0.51 meV of the superconducting substrate (Fig. 2). The spectral intensity (Fig. 2b, f, j) is symmetric with respect to the center of the chain. This is in particular true for the ends of the chains (see below for Fe and Supplementary Note 2 and Supplementary Fig. 2 for Mn). The Fe_20_ chain (Fig. 2b) reveals a zero-energy spectral weight with a maximum localized on the two atoms that terminate the chain, which decays in an oscillatory fashion in intensity toward the center of the chain. Spectra taken at the ends of the chain in comparison to spectra taken at the center of the chain (Fig. 2c, d) show that in addition to this zero-energy spectral weight, also the spectral intensity stemming from YSR bands at a nonzero energy of about +0.1 meV is increased toward the chain’s ends (see arrows in Fig. 2b, c, and d). This reproduces the data of a previous publication, which was taken in a different STM facility using a different STM tip and sample^14^. Note that the spectra in Fig. 2d, h, l are normalized by subtraction of a spectrum averaged over a sufficiently large length along the chain’s interior in order to approximate the difference of the spectral distribution at the chain’s ends with respect to that of an infinite chain. With the help of ab initio and tight-binding model calculations, the zero-energy spectral weight was interpreted as a signature for a Majorana bound state localized at each end of the Fe_20_ chain^14^.

In stark contrast, the Mn_101_ chain’s ends do not show any zero-energy spectral weight (Fig. 2f). In the interior of the chain, there is a YSR band whose energy is slightly smaller than Δ, which is visible as a shoulder of the coherence peak on the negative-bias side (Fig. 2g, h). These results let us conclude that the relatively weak Kondo coupling^23^ of Mn compared to Fe prevents the development of a topologically superconducting phase via the YSR bands^4^. Notably, the energy of the YSR band is slightly decreased at the chain’s ends, and thus somewhat approaches the Fermi level (see arrows in Fig. 2f, g). The changes to the YSR band close to the ends of the Fe chain (see above) and to the YSR band energy close to the ends of the Mn chain could be explained by the reduced coordination number of the chain-terminating atoms. Such changes can, therefore, be reduced by attaching nonmagnetic atoms with a similar orbital structure as the ones in the magnetic chain to both of its ends. Figure 2i–l show that this possibility is provided by chains of Co^hcp^ atoms. The spectral intensity of these chains does not show any change when the tip moves along a line starting from the substrate and then across the entire Co chain. This implies that a close-packed linear chain made from the initially nonmagnetic individual Co^hcp^ atoms on the Re(0001) surface^23^ still has a completely quenched magnetization. The superconductivity from the substrate can, thus, penetrate this chain of atoms. Because both Fe and Co atoms occupy hcp adsorption sites, and because of their identical orbital structure on Re, the Co chain might represent an ideal termination for the Fe chain where the latter shows signatures of a topological superconductor.

### Magnetic properties of Co-terminated Fe chains

**Fig. 3:**
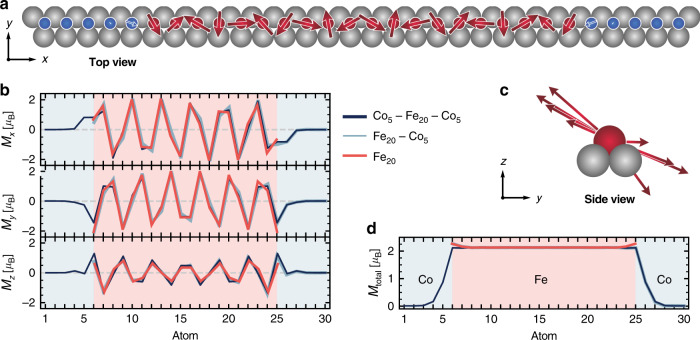
Calculated spin structure of the Fe_20_ chain without and with Co_5_ termination. **a** Top view of the calculated spin structure in the Co_5_–Fe_20_–Co_5_ chain. Blue and red spheres correspond to Co and Fe atoms, respectively. The length of the arrows is proportional to the size of the in-plane component of the magnetic moment at each particular site. **b** *x*, *y*, and *z* components of the magnetic moments in the Co_5_–Fe_20_–Co_5_-, the Fe_20_–Co_5_-, and the Fe_20_ chains. **c** Side view of the Co_5_–Fe_20_–Co_5_ chain cycloidal spin spiral along the negative *x* direction. **d** Total magnetic moment of the atoms along all three chains.

To follow the idea of terminating the magnetic Fe chain by the nonmagnetic Co chain, we first investigate the magnetic properties at the material transition in hybrid Co^hcp^–Fe^hcp^ chains in the normal metallic state of the substrate. This is done via ab initio calculations using the Korringa–Kohn–Rostoker (KKR) Green’s function method based on an embedding scheme, together with an effective spin model (see “Methods” and Supplementary Note 3). Our calculations reveal that the exchange interactions between the nearest and next-nearest neighbors within the Fe_20_ chain are strongly antiferromagnetic (Supplementary Fig. 3), which leads to spin frustration. This frustration is resolved by the formation of a cycloidal spin spiral of wavelength between three and four lattice constants (Fig. 3), in agreement with previous experimental results^14^. Consequently, in this system, spin spirals originate from spin frustration rather than from Dzyaloshinskii–Moriya (DM) interaction. The DM interaction only sets the plane of rotation, which is at an angle of 30° to the surface plane for the Fe_20_ chain (Fig. 3c), and the rotational sense, but it has only a minor effect on the spin-spiral wavelength. Note that for the pure Fe_20_ chain, the magnetic moments and interactions of the Fe atoms at both ends differ from the Fe atoms in the interior of the chain (Fig. 3d and Supplementary Figs. 3 and 4). However, when terminating the Fe_20_ chain with Co_5_ chains, both the magnetic moments and the interactions of these Fe atoms become similar to those of the Fe atoms in the interior of the chain (Fig. 3b, d and Supplementary Figs. 3 and 4). Moreover, the magnetic moments of the Co atoms in the Co_5_ chain attached to the Fe_20_ chain are essentially zero. Only the first Co atom at the transition to the Fe chain has a considerably induced magnetic moment (Fig. 3b, d). As a result, the impact of the Co chains on the magnetic structure in the interior of the Fe chain is negligible. Therefore, already five-atom-long Co chains realize a perfect termination of the Fe_20_ chain with an atomically sharp transition between the Fe chain’s spin-spiral state and the nonmagnetic *d* states of the Co chain.

### In-gap electronic structure of Co-terminated Fe chains

**Fig. 4:**
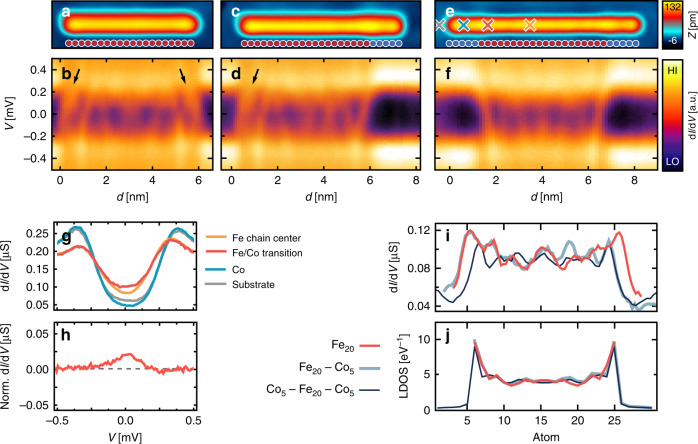
In-gap states of the Fe_20_ chains without and with Co_5_ termination. **a** Constant-current scanning tunneling microscope (STM) image of the Fe_20_ chain. **b** Differential tunneling conductance along the Fe_20_ chain aligned with the topography in (**a**). **c** Constant-current STM image of the Fe_20_–Co_5_ chain. **d** Differential tunneling conductance along the Fe_20_–Co_5_ chain. **e** Constant-current STM image of the Co_5_–Fe_20_–Co_5_ chain. **f** Differential tunneling conductance along the Co_5_–Fe_20_–Co_5_ chain. Arrows in (**b**, **d**): see text. **g** d*I*/d*V* spectra of the Co_5_–Fe_20_–Co_5_ chain taken at the positions marked by crosses in (**e**) (*V*_stab_ = 1 mV, *I*_stab_ = 0.2 nA, and *V*_mod_ = 20 µV). **h** d*I*/d*V* spectrum from (**g**) taken at the Fe/Co transition and normalized by subtraction of a spectrum averaged along the chain’s interior. **i** Experimental zero-bias d*I*/d*V* signal along the three different chains as indicated left of (**j**) extracted from (**b**, **d**, **f**). **j** Spectral weight at the Fermi energy along the three different chains as indicated on the left, calculated from the tight-binding model. The tight-binding model parameters, except for Δ, are extracted from the ab initio calculations (“Methods” and Supplementary Notes 4 and 5). Δ is chosen as to reproduce the experimentally observed spatial decay of the zero-bias spectral weight from the termination of the spin chain toward the chain center.

Keeping these results in mind, we experimentally investigate the in-gap electronic structure in the superconducting state of the substrate along hybrid Fe_20_–Co_5_ and Co_5_–Fe_20_–Co_5_ chains that have been built by successively attaching Co atoms first to the right (Fig. 4c, d) and then to the left side (Fig. 4e, f) of the pure Fe_20_ chain (Fig. 4a, b). Indeed, the in-gap electronic structure measured on the last few Fe atoms close to the Co termination is considerably different from those measured on the last few Fe atoms at the open ends in the hybrid chains and in the pure Fe_20_ chain. In particular, the spectral intensity of the YSR band at +0.1 meV, which is increased at the open ends of the pure Fe_20_ chain and at the open end of the Fe_20_–Co_5_ chain (see arrows in Fig. 4b, d), is almost completely moved out of the gap region, both at the single and at the two Co-terminated ends of the Fe_20_–Co_5_ and Co_5_–Fe_20_–Co_5_ chains, respectively (see also the spectra in Fig. 4g, h for comparison with Fig. 2c, d). Most notably, the zero-energy spectral weight maximum is persistent at the Co-terminated Fe chains. Its position is only slightly shifted toward the interior of the Fe part of the hybrid chain by about two atomic lattice constants after having attached the Co termination. This is visible in the zero-energy spectral weight extracted from Fig. 4b, d, f as shown in Fig. 4i. Simultaneously, the five local maxima and minima of the oscillations of the zero-energy spectral weight in the interior of the chain shift slightly toward the center. This experimental observation is consistent with the interpretation of the zero-energy spectral weight as a signature of a Majorana bound state localized at each end of the Fe_20_ chain, which is expected to be protected against the local perturbation of the Fe_20_ chain by the Co termination. Such a perturbation, which is not affecting the internal spin structure of the Fe_20_ chain, can merely shift the lateral position of a Majorana bound state, but cannot completely remove it. In contrast, it can strongly influence the topologically trivial YSR bands. In order to further support this interpretation, we also investigated the topological properties of infinite Fe chains and the spatially resolved in-gap electronic structure of the pure and Co-terminated chains with a tight-binding model using the parameters extracted from the above KKR calculations (see “Methods” and Supplementary Notes 4 and 5). For an appropriately tuned superconducting energy gap Δ, the tight-binding model reproduces the zero-energy spectral weight localized at both ends of the pure Fe chains, whose spatial localization is slightly increased when attaching the Co terminations, as shown in Fig. 4j. Using the same Δ, the tight-binding model for the infinite Fe chain displays the topologically superconducting phase. These results corroborate that the experimentally observed zero-energy spectral weight localized at the ends of the Fe chain is a signature of a Majorana bound state that persists when terminating the chain with topologically trivial Co chains.

Our results, thus, suggest that appropriate terminations of topologically superconducting chains realized by artificial hybrid transition-metal atom chains can be used to tune the properties of Majorana bound states and trivial YSR bands. We thereby establish essential next steps toward the atom-by-atom design of hybrid networks of spin chains and nonmagnetic superconducting chains, and toward the controlled manipulation of Majorana bound states, which are desired for Majorana braiding and the demonstration of topological quantum computation.

## Methods

### Experimental procedures

All measurements were performed in a home-built ultra-high-vacuum STM setup at *T* = 0.3 K^24^. We used electrochemically etched tungsten tips that were flashed to *T* = 1500 K before inserting them into the STM. The Re(0001) crystal was cleaned by Ar ion sputtering, followed by multiple cycles of O_2_ annealing at *T* = 1530 K and flashing to *T* = 1800 K. Mn, Fe, and Co atoms were successively deposited keeping the substrate at *T* < 10 K. The bias-dependent differential tunneling conductance d*I*/d*V* was measured using a Lock-In amplifier by modulating the bias voltage *V* with *V*_mod_ = 20–40 µV at a frequency of *f*_mod_ = 4142 kHz, and at constant tip height stabilized at a bias voltage *V*_stab_ and tunnel current *I*_stab_ before opening the feedback loop for measurement. The bias voltage is applied to the sample and zero bias corresponds to *E*_F_. Single atoms were manipulated using STM-tip induced atom manipulation by lowering the bias voltage and increasing the setpoint current to the manipulation parameters *V* = 1 mV and *I* = 100 nA.

### Ab initio calculations

The density functional theory (DFT) calculations were performed employing the full-potential KKR Green function method with spin–orbit coupling added to the scalar relativistic approximation (see Supplementary Note 3)^25^. The exchange and correlation potential is treated within the local spin-density approximation using the parameterization of Vosko, Wilk, and Nusair^26^. The Re(0001) substrate is modeled by 22 layers of Re augmented by two vacuum regions corresponding to four interlayer distances. A k-mesh of 150 × 150 and an angular momentum cutoff for the scattering problem of *l*_max_ = 3 are used. The magnetic chains are deposited in the hcp-stacking position on the Re(0001) surface with a relaxation of 20% of the interlayer distance toward the Re surface, using an embedding technique. Three systems are investigated: in addition to an Fe_20_ chain, we added five Co atoms to one end of the chain (Fe_20_–Co_5_) and five Co atoms to each end of the chain (Co_5_–Fe_20_–Co_5_). The real-space clusters that are embedded on the Re(0001) surface contain the nearest-neighbor Re atoms (and vacuum sites), resulting in cluster sizes of 146, 181, and 216 sites, respectively. The magnetic exchange interactions were obtained using the magnetic force theorem in the frozen-potential approximation and the infinitesimal rotation method^27,28^. The site-resolved magnetic on-site anisotropy is obtained using the method of constraining fields^29^.

### Effective spin-model calculations

The magnetic exchange interactions and the on-site magnetic anisotropy obtained from the ab initio calculations are used to parameterize the following classical Heisenberg model (see Supplementary Note 3):

1 $$\begin{array}{*{20}{c}} {{\cal{H}} = \mathop {\sum}\limits_i {{\boldsymbol{e}}_i} \overline{\overline {\cal{K}}} _i{\boldsymbol{e}}_i + \frac{1}{2}\mathop {\sum}\limits_{ij} {J_{ij}} {\boldsymbol{e}}_i \cdot {\boldsymbol{e}}_j + \frac{1}{2}\mathop {\sum}\limits_{ij} {{\boldsymbol{D}}_{ij}} \cdot \left( {{\boldsymbol{e}}_i \times {\boldsymbol{e}}_j} \right) + \frac{1}{2}\mathop {\sum}\limits_{ij} {{\boldsymbol{e}}_i} \overline{\overline J} _{ij}^{{\mathrm{sym}}}{\boldsymbol{e}}_j,} \end{array}$$

where the unit vectors ***e***_*i*_ point along the magnetization of atom *i* in the chain, $\overline{\overline {\cal{K}}} _i$ are the magnetic on-site anisotropy matrices, *J*_*ij*_ are the isotropic exchange interactions, ***D***_*ij*_ are the Dzyaloshinskii–Moriya interaction vectors, and $\overline{\overline J} _{ij}^{s{\mathrm{ym}}}$ are the symmetric anisotropic exchange interaction matrices. The magnetic ground states are obtained from numerically minimizing the Heisenberg model starting from several random initial configurations of ***e***_*i*_.

### Tight-binding model calculations

The finite magnetic chains on superconducting Re(0001) are modeled by a tight-binding Hamiltonian with realistic parameters obtained from DFT (see Supplementary Notes 4 and 5). The model explicitly considers the *d* orbitals of the chain atoms, with parameters that account for the Re substrate via an effective Hamiltonian construction. The site-resolved on-site electronic structure is modeled by a chemical potential, a spin splitting generating the magnetic moments, a local spin–orbit coupling, and an orbital-dependent crystal-field splitting. The hoppings between the sites are assumed to be hermitian, spin-independent, and symmetric in the orbitals. Superconductivity is added in the *s*-wave approximation with the local pairing potential being an orbital-independent parameter^30^. The local density of states is obtained by assuming an artificial temperature broadening, which is half of the spectral gap of the superconducting state.

## Supplementary information

Supplementary Information

## Data Availability

The authors declare that the data supporting the findings of this study are available within the paper and its supplementary information files.
