# Supplementary material for: Controlling in-gap end states by linking nonmagnetic atoms and artificially-constructed spin chains on superconductors
Source: Nat Commun. 2020 Sep 18;11:4707. doi: 10.1038/s41467-020-18540-3 (PMC7501864; doi:10.1038/s41467-020-18540-3)
Supplement: Supplementary file 1 — Supplementary Information [file 41467_2020_18540_MOESM1_ESM.pdf]

Supplementary Information

Controlling in-gap end states by linking nonmagnetic atoms and artificially-constructed spin chains on superconductors

Schneider *et al.*

Supplementary Note 1 | Determining the geometric structure of the Mn chains on Re(0001)

It was shown that single Mn atoms on a Re(0001) surface are only stable on fcc sites[1]. When constructing artificial closed packed chains of Mn atoms, however, it turns out that the atoms in the chain occupy both fcc and hcp positions. This can be seen in Supplementary Fig. 1a, where the lattice of surface Re atoms was overlayed on a constant-current STM image of a Mn_6 chain surrounded by four single Mn^{fcc} atoms. It is clearly visible that all single atoms occupy the same type of adsorption site. In contrast, the chain topography can only be explained if neighboring atoms are alternating between fcc and hcp adsorption sites. This is similarly valid for a Mn_9 chain shown in Supplementary Fig. 1b, where three of the four single atoms were attached to the chain of Supplementary Fig. 1a.

With a sharper tip, the internal zig-zag structure of the Mn chains can be directly visualized. Supplementary Fig. 1c shows three chains of 5, 6 and 7 Mn atoms, respectively, displaying a clear zig-zag pattern. The internal structure of the chains is sketched below each STM image. Moreover, the alternation of adsorption sites can be seen in the apparent tilt of the chain's ends: for odd numbered chains, the right chain end in Supplementary Fig. 1c is pointing upwards whereas it points downwards for the even numbered chains. This effect is also faintly visible with blunter tips as, e.g., in Supplementary Fig. 1a,b.

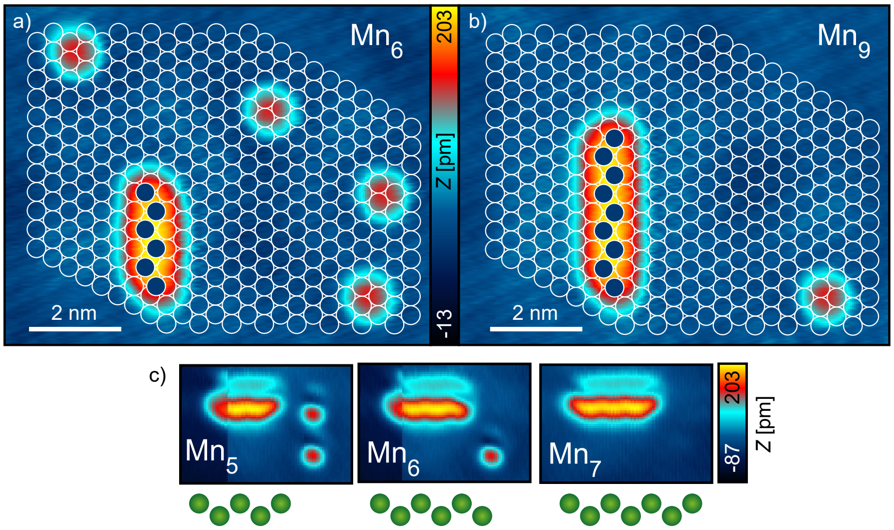

Supplementary Figure 1 | Geometric structure of the zig-zag Mn chains. (**a**) Constant-current STM image of four single Mn^{fcc} atoms and a zig-zag chain consisting of 6 Mn atoms. The white circles indicate the positions of the surface Re atoms. (**b**) Constant-current STM image of one single Mn^{fcc} atom and a zig-zag chain consisting of 9 Mn atoms. (**c**) Constant-current STM images of chains consisting of 5, 6 and 7 Mn atoms recorded with a sharper tip. The atomic positions in the chains are sketched below the STM images. All images (**a**-**c**) are recorded with $V = 10\,\text{mV}$, $I = 0.5\,\text{nA}$.

Supplementary Note 2 | Symmetry of the spectral weights

The spectral intensity measured along the artificially constructed Fe chains exhibits mirror symmetry with respect to the chain's center, as shown in Fig. 4b of the main manuscript. This symmetry is also found for Mn chains as long as 101 atoms (Supplementary Fig. 2a). As can be seen in Supplementary Fig. 2b, both ends of the chain are showing the same end state at $V \approx 200\,\mu$V. This proves a remarkable reproducibility of the experiments.

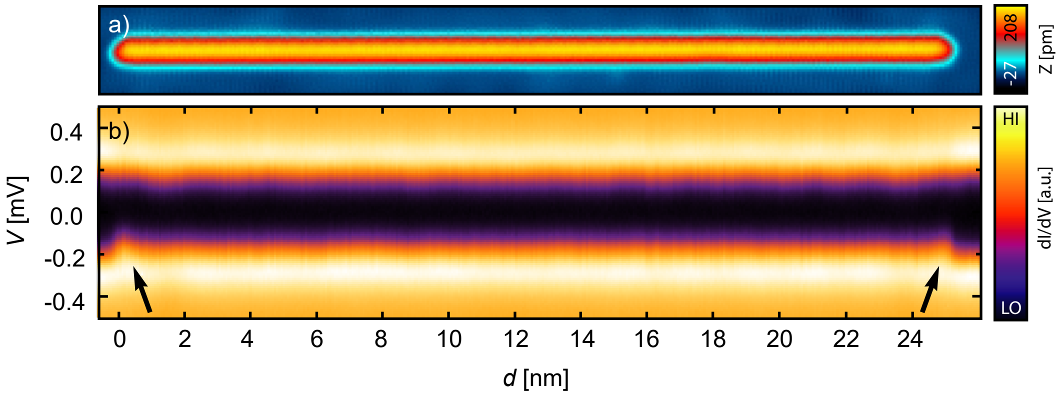

Supplementary Figure 2 | Spectroscopy along the entire Mn_{101} chain. (**a**) Constant-current STM image of a zig-zag chain consisting of 101 Mn atoms recorded with $V = 6\,$mV, $I = 0.2\,$nA. (**b**) Differential tunneling conductance along the Mn_{101} chain aligned with the topography in (**a**). $V_{\mathrm{stab}} = 1\,$mV, $I_{\mathrm{stab}} = 0.5\,$nA, $V_{\mathrm{mod}} = 40\,\mu$V.

Supplementary Note 3 | Magnetic interactions

The spin system is modeled by a classical Heisenberg model of the following form

$$\mathcal{H} = \sum_i \mathbf{e}_i \bar{\bar{\mathcal{K}}}_i \mathbf{e}_i + \frac{1}{2}\sum_{ij} J_{ij}\mathbf{e}_i \cdot \mathbf{e}_j + \frac{1}{2}\sum_{ij} \mathbf{D}_{ij} \cdot (\mathbf{e}_i \times \mathbf{e}_j) + \frac{1}{2}\sum_{ij} \mathbf{e}_i \bar{\bar{J}}_{ij}^{\text{sym}} \mathbf{e}_j \quad , \tag{1}$$

with $\mathbf{e}_i$ being the orientation of the magnetic moment of atom i, and including the on-site anisotropy $\mathcal{K}_i$ of atom i and all magnetic exchange interactions up to the bilinear level, namely the isotropic exchange interaction J_{ij}, the Dzyaloshinskii-Moriya interaction (DMI) $\mathbf{D}_{ij}$, and the symmetric anisotropic exchange interaction J_{ij}^{sym} between atom i and j. Supplementary Fig. 3 shows the isotropic magnetic exchange interaction for the three different chains obtained from the infinitesimal rotation method [2,3]. The nearest neighbor exchange as well as the next nearest neighbor exchange are shown. Both indicate a strong antiferromagnetic coupling, which leads to a frustrated ground state. The frustration gives rise to a spin spiral of wavelength between three and four lattice constants. Supplementary Fig. 4 shows the computed DMI for the chains [3]. The DMI sets the plane of rotation and the rotational sense. Due to its strength it has only minor effects on the spin spiral wavelength. For both interactions one can see that adding Co to the Fe chain reduces the boundary effects in the Fe chain.

The magnetic on-site anisotropy is traceless by convention and is obtained using the method of constraining fields [4]. The chains are set to the ferromagnetic state, which is rotated along the path $z \to x \to y \to z$ using 21 orientations. For each configuration magnetic constraining fields are used to self-consistently stabilize the ferromagnetic state. Combining the constraining fields and the known magnetic exchange interactions obtained from the infinitesimal rotation method, the site-resolved on-site anisotropy is obtained by using a least squares fit. The anisotropy of the central Fe atom is found to be

$$\bar{\bar{\mathcal{K}}}_{15} = \begin{pmatrix} 0.13 & 0.00 & 0.00 \\ 0.00 & 0.21 & -0.65 \\ 0.00 & -0.65 & -0.34 \end{pmatrix} \text{meV} \quad , \tag{2}$$

with the eigensystem ($\bar{\bar{\mathcal{K}}}\mathbf{n} = \lambda\mathbf{n}$)

$$\lambda = \{-0.77, 0.13, 0.64\} \quad \text{with} \quad \mathbf{n} = \left\{ \begin{pmatrix} 0.0 \\ 0.54 \\ 0.84 \end{pmatrix}, \begin{pmatrix} 1.0 \\ 0.0 \\ 0.0 \end{pmatrix}, \begin{pmatrix} 0.0 \\ -0.84 \\ 0.54 \end{pmatrix} \right\} \quad . \tag{3}$$

This on-site anisotropy is of biaxial type, with an easy-axis lying in the yz-plane with a polar angle of $32°$.

The rightmost magnetic interaction considered in Supplementary Equation (1) is the symmetric anisotropic exchange interaction, which for the central bond of the Co terminated Fe chain

is

$$\bar{\bar{J}}^{\text{sym}}_{14,15} = \begin{pmatrix} -0.39 & 0.00 & 0.00 \\ 0.00 & -0.68 & 0.30 \\ 0.00 & 0.30 & 1.08 \end{pmatrix} \text{ meV} \quad , \tag{4}$$

while for the central next-nearest neighbor bond it is given by

$$\bar{\bar{J}}^{\text{sym}}_{14,16} = \begin{pmatrix} 0.50 & 0.00 & -0.01 \\ 0.00 & 0.79 & 0.50 \\ -0.01 & 0.50 & -1.28 \end{pmatrix} \text{ meV} \quad . \tag{5}$$

We see that these two contributions tend to counteract each other. Both the on-site anisotropy and the symmetric anisotropic exchange interaction exhibit boundary effects, which are modulated if

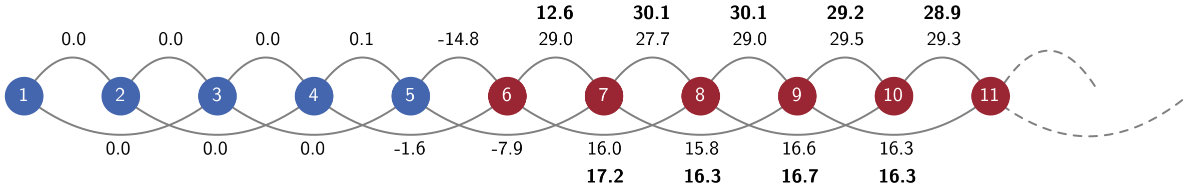

Supplementary Figure 3 | Isotropic exchange interaction (J_{ij}) at the Co-Fe transition. Exchange interactions between Co (blue circles) and Fe atoms (red circles). The bold values show the exchange in the pure Fe_{20} chain. The exchange in the Co_5-Fe_{20} chain is shown in regular font. The three Fe atoms next to the Co are seen to be substantially affected by attaching the Co.

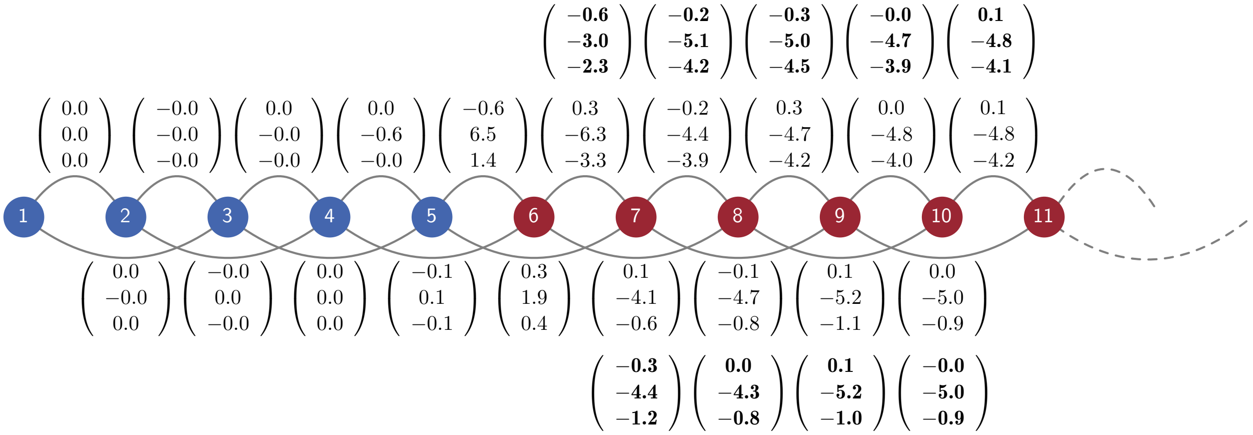

Supplementary Figure 4 | Dzyaloshinskii-Moriya interaction (D_{ij}) at the Co-Fe transition. Each vector contains the components of the interaction from the atom on the left to the atom on the right, $\mathbf{D} = (D_x, D_y, D_z)$. Co atoms are marked by blue circles and Fe atoms by red circles. The bold values show the exchange in the pure Fe_{20} chain. The exchange in the Co_5-Fe_{20} chain is shown in regular font.

the Fe chain is terminated by Co. However, due to the overall weakness of these interactions when compared to the DMI and the isotropic exchange, they play only a minor role for the obtained magnetic structures. In fact, the obtained ground state magnetic configuration can be explained solely with the DMI and the isotropic exchange, with the symmetric anisotropic contributions deciding the details of the spin spiral rotation with respect to the crystal directions.

Supplementary Note 4 | Tight-binding parameters

To obtain a tight-binding model for the d-orbitals of the magnetic chain that accurately describes the electronic structure near the Fermi energy (important for superconductivity) and takes into account the strong coupling to the Re(0001) substrate, we partition the KKR Green function into blocks pertaining to the chain and those pertaining to the substrate, and define an effective Hamiltonian as follows:

$$G(E) = (E - \mathcal{H})^{-1} \quad \Longrightarrow \quad \mathcal{H}_{\text{eff}} \approx E_{\text{F}} - G^{-1}_{\text{chain,chain}}(E_{\text{F}}) \quad . \tag{6}$$

The effective tight-binding parametrization can be split into an on-site and a hopping part,

$$\mathcal{H}_{\text{chain}} = \sum_i \mathcal{H}_i^{\text{on-site}} + \mathcal{H}_{\text{hopping}} \quad . \tag{7}$$

The on-site Hamiltonian of atom i (omitting the atom index in the following) is given by

$$\begin{aligned} \mathcal{H}^{\text{on}-\text{site}} = \sum_{mm'} \sum_{ss'} \big(& E_d \, \delta_{mm'} \delta_{ss'} + U \, \mathbf{m} \cdot \boldsymbol{\sigma}_{ss'} \delta_{mm'} + \lambda \, \mathbf{L}_{mm'} \cdot \boldsymbol{\sigma}_{ss'} + C^{(\text{re})}_{mm'} \delta_{ss'} \\ & + \mathrm{i}\Gamma \, \delta_{mm'} \delta_{ss'} + \mathrm{i} C^{(\text{im})}_{mm'} \delta_{ss'} \big) c^\dagger_{ims} c_{im's'} \quad , \end{aligned} \tag{8}$$

where E_d is the average energy of the d-orbitals with respect to the Fermi energy, U represents the spin splitting when the local magnetic moment is set to the $\mathbf{m}$ direction, $\boldsymbol{\sigma} = (\sigma_x, \sigma_y, \sigma_z)$ is the vector of Pauli matrices, λ is the strength of the local spin-orbit coupling, $\mathbf{L}$ is the local orbital angular momentum operator, $C^{(\text{re})}$ is an orbital dependent energy shift corresponding to the crystal field splitting, and Γ as well as $C^{(\text{im})}$ are non-hermitian contributions that result from the hybridization with the substrate, which we will not consider in the model including superconductivity.
The hopping Hamiltonian is given by

$$\mathcal{H}_{\text{hopping}} = \sum_{ij} \sum_{mm'} \sum_{ss'} t_{ims,jm's'} c^\dagger_{ims} c_{jm's'} \quad . \tag{9}$$

Since the Hamiltonian is hermitian the hoppings have to fulfil $t_{ims,jm's'} = t_{jm's',ims}$. In addition, we assume the hopping to be spin-independent and symmetric in the orbitals, $t_{ims,jm's'} = t_{ims,jm's}\delta_{ss'} = t_{im's,jms}\delta_{ss'}$.
The on-site parameters of the Fe_{20} and the Co_5-Fe_{20}-Co_5 chains are shown in Supplementary Tables 1 and 3, respectively. The Co termination has only a minor effect on the Fe chain. The chemical potential, the hybridization and the spin-orbit coupling are unaffected, while the spin

splitting decreases. Interestingly, the Co chain is quite similar to the Fe chain in terms of the on-site quantities. Only the spin splitting decreases according to the magnetic moment. The crystal field splitting $\Delta^{(\mathrm{re})}$ of one of the central Fe atoms of the Fe_{20} chain is shown in Supplementary Table 2. The Co terminations have only minor effects on the crystal field matrix with deviation of less than $0.1\,\mathrm{eV}$. The nearest neighbor hoppings between Co atoms are also comparable to those between Fe atoms, as shown in Supplementary Tables 4 and 5.

To set up an effective tight-binding model for an infinite chain, we use the parametrization of the center of the Fe_{20} chain, including hoppings up to the 4th neighbor.

	E_d	U	Γ	λ
Fe 1	-0.884	1.090	-0.750	0.041
Fe 2	-0.931	1.057	-0.776	0.042
Fe 3	-0.901	1.034	-0.775	0.042
Fe 4	-0.909	1.040	-0.774	0.041
Fe 5	-0.908	1.038	-0.773	0.042
Fe 6	-0.911	1.040	-0.773	0.041
Fe 7	-0.911	1.039	-0.774	0.042
Fe 8	-0.910	1.039	-0.775	0.042
Fe 9	-0.910	1.039	-0.774	0.042
Fe 10	-0.910	1.039	-0.774	0.042
Fe 11	-0.910	1.039	-0.774	0.042
Fe 12	-0.910	1.039	-0.774	0.042
Fe 13	-0.910	1.039	-0.775	0.042
Fe 14	-0.911	1.039	-0.774	0.042
Fe 15	-0.911	1.040	-0.773	0.041
Fe 16	-0.908	1.038	-0.773	0.042
Fe 17	-0.909	1.040	-0.774	0.041
Fe 18	-0.901	1.034	-0.775	0.042
Fe 19	-0.931	1.057	-0.776	0.042
Fe 20	-0.884	1.090	-0.750	0.041

Supplementary Table 1 | On-site parameters for the Fe_{20} chain. All data given in units of eV.

	xy	yz	z^2	xz	x^2-y^2
xy	-0.287	0.000	0.000	0.564	0.000
yz	0.000	-0.208	-0.004	0.000	0.551
z^2	0.000	-0.004	1.025	0.000	-0.020
xz	0.564	0.000	0.000	-0.277	0.000
x^2-y^2	0.000	0.551	-0.020	0.000	-0.253

Supplementary Table 2 | Crystal field splitting for the Fe_{20} chain. $C^{(\mathrm{re})}$ of the central Fe atom in the Fe_{20} chain. All the data is given in units of eV.

	E_d	U	Γ	λ
Co 1	-0.874	0.020	-0.625	0.038
Co 2	-0.933	0.049	-0.648	0.039
Co 3	-0.935	0.192	-0.649	0.039
Co 4	-0.939	0.246	-0.650	0.039
Co 5	-1.001	0.416	-0.654	0.039
Fe 6	-0.882	1.019	-0.769	0.042
Fe 7	-0.893	1.029	-0.771	0.042
Fe 8	-0.911	1.039	-0.775	0.042
Fe 9	-0.912	1.039	-0.774	0.041
Fe 10	-0.908	1.037	-0.774	0.042
Fe 11	-0.911	1.039	-0.774	0.041
Fe 12	-0.910	1.040	-0.774	0.042
Fe 13	-0.910	1.039	-0.774	0.042
Fe 14	-0.910	1.039	-0.774	0.041
Fe 15	-0.910	1.039	-0.774	0.041
Fe 16	-0.910	1.039	-0.774	0.041
Fe 17	-0.910	1.039	-0.774	0.041
Fe 18	-0.910	1.039	-0.774	0.042
Fe 19	-0.910	1.040	-0.774	0.042
Fe 20	-0.911	1.039	-0.774	0.041
Fe 21	-0.908	1.037	-0.774	0.042
Fe 22	-0.912	1.039	-0.774	0.041
Fe 23	-0.911	1.039	-0.775	0.042
Fe 24	-0.893	1.029	-0.771	0.042
Fe 25	-0.882	1.019	-0.769	0.042
Co 26	-1.001	0.416	-0.654	0.039
Co 27	-0.939	0.247	-0.650	0.039
Co 28	-0.935	0.192	-0.649	0.039
Co 29	-0.933	0.050	-0.648	0.039
Co 30	-0.874	0.020	-0.625	0.038

Supplementary Table 3 | On-site parameters for the Co_5-Fe_{20}-Co_5 chain. All the data is given in units of eV.

	xy	yz	z^2	xz	x^2-y^2
xy	0.289	0.003	-0.002	-0.066	-0.000
yz	0.003	0.002	0.004	0.004	0.101
z^2	-0.002	0.004	-0.071	-0.007	0.262
xz	-0.066	0.004	-0.007	0.036	0.002
x^2-y^2	-0.000	0.101	0.262	0.002	-0.322

Supplementary Table 4 | Hoppings between the first two neighboring Co atoms at the transition in the Co_5-Fe_{20}-Co_5 chain. All the data is given in units of eV.

	xy	yz	z^2	xz	x^2-y^2
xy	0.348	0.000	-0.000	-0.078	-0.000
yz	0.000	0.001	0.002	-0.000	0.121
z^2	-0.000	0.002	-0.128	-0.000	0.307
xz	-0.078	-0.000	-0.000	0.048	-0.000
x^2-y^2	-0.000	0.121	0.307	-0.000	-0.379

Supplementary Table 5 | Hoppings between two neighboring Fe atoms in the center of the Co_5-Fe_{20}-Co_5 chain. All the data is given in units of eV.

Supplementary Note 5 | Superconductivity in the tight-binding model

Superconductivity is added to the tight-binding model in the s-wave approximation,

$$\mathcal{H} = \mathcal{H}_{\text{chain}} + \Delta \sum_i \sum_m \left(c_{im\downarrow} c_{im\uparrow} + c^{\dagger}_{im\uparrow} c^{\dagger}_{im\downarrow} \right) \quad , \tag{10}$$

where the pairing potential Δ, which couples electrons and holes, represents s-wave superconductivity, and is chosen to be local and orbital independent [5]. The Bogoliubov-de Gennes (BdG) Hamiltonian is given by

$$\mathcal{H} = \begin{pmatrix} \mathcal{H}_{\text{chain}} & \Delta \, \mathbb{1} \otimes \mathrm{i}\sigma_y \\ -\Delta \, \mathbb{1} \otimes \mathrm{i}\sigma_y & -\mathcal{H}^{*}_{\text{chain}} \end{pmatrix} \quad , \tag{11}$$

where the basis $\Psi = (u_{\uparrow}, u_{\downarrow}, v_{\uparrow}, v_{\downarrow})$ with u corresponding to the particle wavefunction and v to the hole wavefunction is used. For each eigenstate n at energy E_n there is a corresponding eigenstate n' at energy $E_{n'} = -E_n$ ensuring the particle hole symmetry in the superconducting state.
The band structure obtained using the realistic tight-binding parameters from DFT is shown in Supplementary Fig. 5. A spin spiral rotating in a plane making an angle of 30° with the xy-plane (see magnetic structure obtained from the Heisenberg model in the main text) with a wavelength of four lattice constants is assumed, which is based on the experimental observation [6] and the theoretical prediction of a wavelength of approximately 3.25 lattice constants. A superconducting pairing potential of $\Delta = 0.83\,\text{eV}$ was used, which gives rise to an enormous gap (unrealistic), but is needed to match the localization of the zero energy bound states found in experiment. This is a drawback of this particular model, rising from the fact that we add the proximity-induced superconducting pairing potential as a parameter and not via an interaction with a superconducting host [5]. With this set of parameters, the Majorana number computed via the Pfaffians of the Hamiltonian in the Majorana basis, Pf(A), indicates that the chain is in the non-trivial phase.

The local density of states (LDOS) of site i at the Fermi level is calculated using the eigenstates $|n\rangle$ and the corresponding eigenenergies E_n of the BdG Hamiltonian, Supplementary Equation (11),

$$\rho_i(E = 0) = \frac{1}{\pi} \sum_n \langle n | \mathcal{P}_i | n \rangle \; \frac{\eta}{E_n^2 + \eta^2} \quad , \tag{12}$$

where $\mathcal{P}_i$ projects the particle part of the eigenstates on site i and η is an energy broadening responsible for the Lorentzian smearing. Supplementary Fig. 6 shows the local density of states using the DFT parametrization and the magnetic structure obtained from the Heisenberg model as function of the energy broadening (Supplementary Fig. 6a) and for a specific energy broadening,

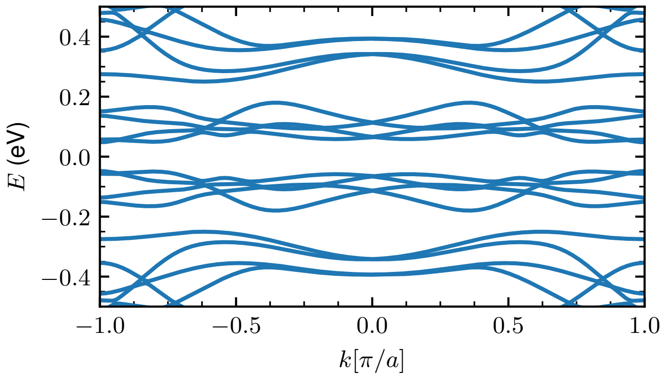

Supplementary Figure 5 | Band structure of the pure Fe chain. The band structure was calculated using an effective Hamiltonian from DFT (see equation (10)). The pairing potential is set to $\Delta = 0.83\,\mathrm{eV}$. Hoppings are included up to the 4th neighbor. The magnetic moments are assumed to rotate in the plane that makes an angle of $30°$ with the xy-plane with a wavelength of four lattice constants. The parameters are taken from the center of the Fe_{20} chain. The Majorana number of $\mathcal{M} = -1$ indicates that the chain is in the non-trivial phase.

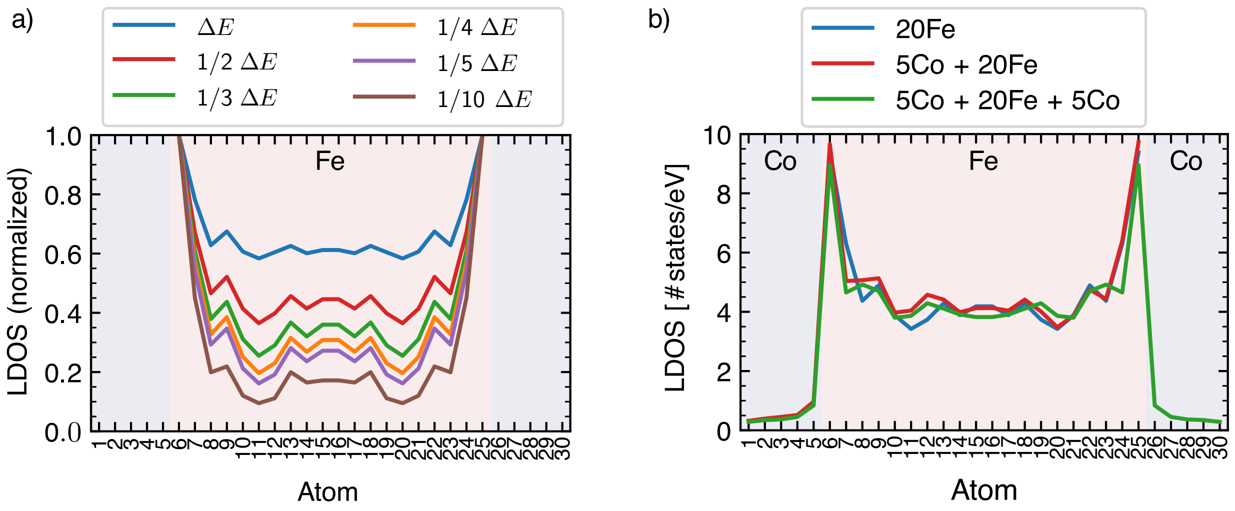

Supplementary Figure 6 | Local density of states of the different magnetic chains. The effective Hamiltonians are taken from DFT. The pairing potential is set to $\Delta = 0.83\,\mathrm{eV}$ for which the infinite Fe chain is in the non-trivial phase with a Majorana number of -1 and a band gap of $\Delta E = 0.09\,\mathrm{eV}$ (see Supplementary Fig. 5). The magnetic structures are obtained from minimizing a Heisenberg model with DFT input parameters (see main text). a) Dependence of the density of states at the Fermi level of the Fe_{20} chain on the energy broadening. b) Local density of states at the Fermi level for a broadening of $1/2\Delta E$ for the three different chains.

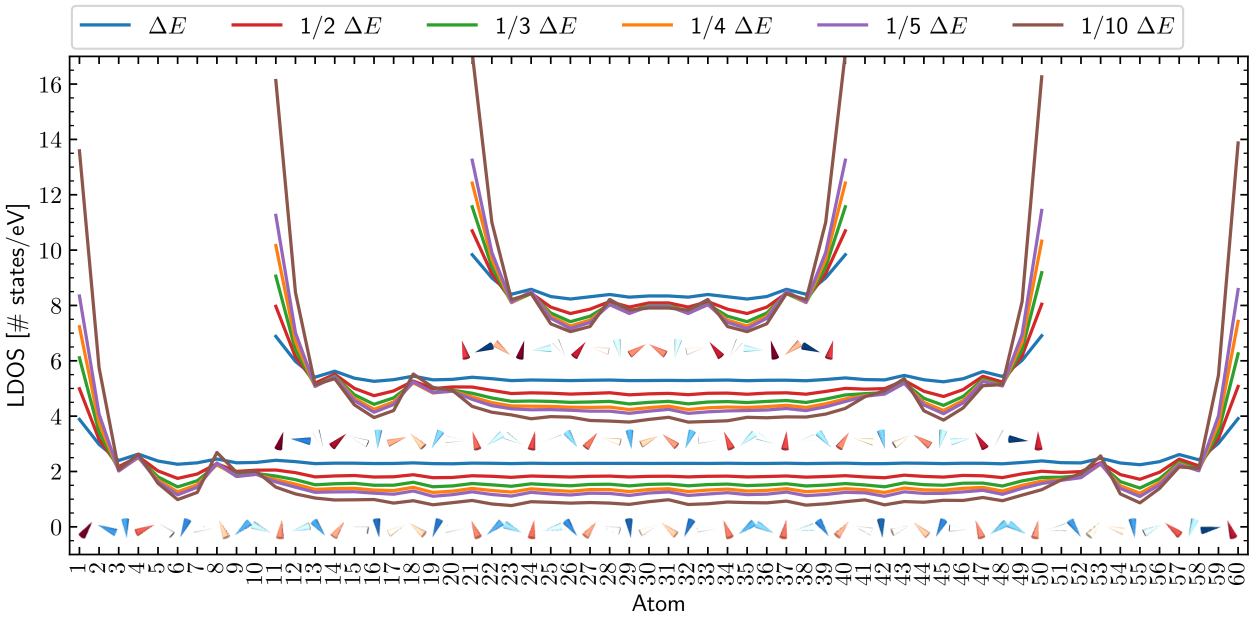

Supplementary Figure 7 | Local density of states of three Fe chains of different length (20, 40 and 60 atoms) as function of the energy broadening. ΔE is the estimated superconducting gap of the infinite Fe chain (see Supplementary Figure 5). The effective Hamiltonian of the Fe_{20} chain taken from DFT is used as a reference to construct the Hamiltonians of the Fe_{40} and Fe_{60} chains by duplicating the electronic properties of the central Fe atoms. The inset cones indicate the magnetic structures of the different chains obtained from minimizing a bilinear Heisenberg model (see Supplementary Note 3). The curves of the Fe_{20} and Fe_{40} chains are shifted for a better visualization.

which is half of the found band gap of the infinite chain (Supplementary Fig. 6b). All chains show a distinct boundary state in the Fe chain.

To analyze the stability of the boundary states with respect to the number of Fe and Co atoms in the chains, we performed additional simulations. First, we extended the pure Fe chains by duplicating the tight-binding parameters and the magnetic exchange interactions of the central Fe atoms in the calculated Fe_{20} chain to construct a 40- and 60-atomic Fe chain. Supplementary Fig. 7 shows the resulting local density of states computed at zero energy and for different energy broadenings. As can be seen, the boundary states are robust and not affected by the length of the Fe chain. Supplementary Fig. 8 shows a similar construction for different numbers of Co atoms attached to the Fe chain. For those calculations, the magnetic profile was kept fixed, while the tight-binding model uses duplicated parameters of the central Co atom taken from the Fe_{20} + Co_5 calculation. Also, this simulation indicates that the states in the Fe chain are robust against the number of Co atoms attached to the Fe chain.

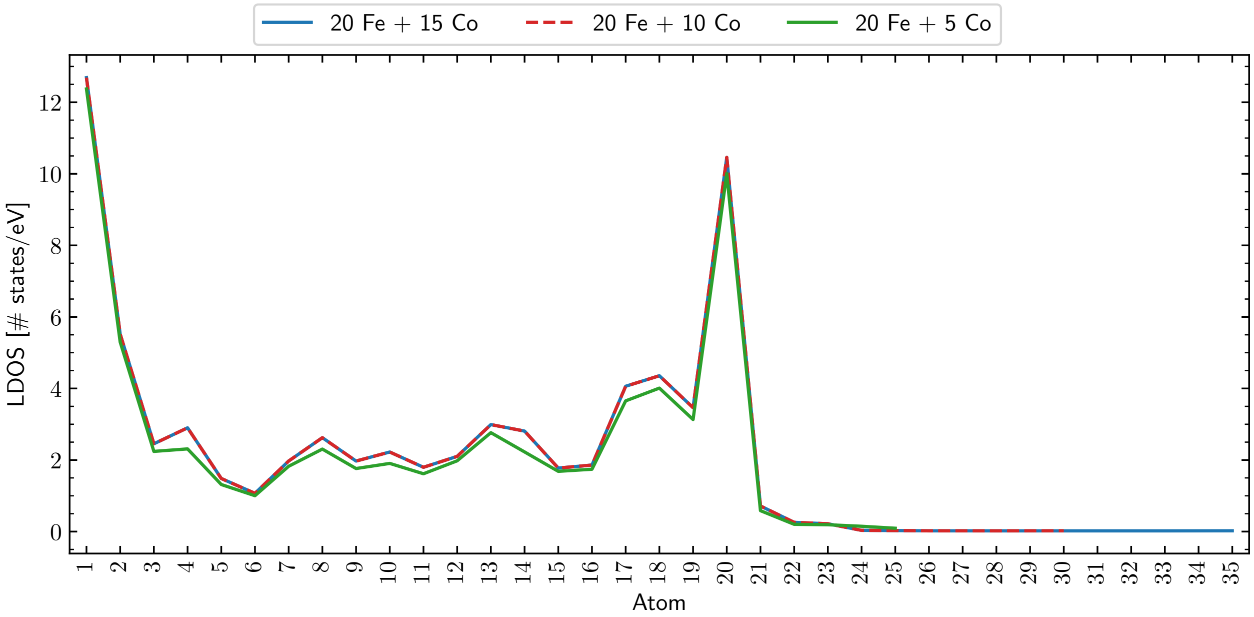

Supplementary Figure 8 | Local density of states of the Fe_{20} chain with 5, 10, and 15 Co atoms attached to one side of the chain. The energy broadening is set to $1/10\Delta E$. To extend the Fe_{20}+Co_5 chain the electronic properites of the central Co atom is duplicated, while the magnetic profile is kept fixed.

Supplementary References

1. Schneider, L. *et al.* Magnetism and in-gap states of 3d transition metal atoms on superconducting Re. *npj Quantum Materials* **4**, 42 (2019).

2. Liechtenstein, A. I., Katsnelson, M. I., Antropov, V. P. & Gubanov, V. A. Local spin density functional approach to the theory of exchange interactions in ferromagnetic metals and alloys. *Journal of Magnetism and Magnetic Materials* **67**, 65 (1987).

3. Ebert, H. & Mankovsky, S. Anisotropic exchange coupling in diluted magnetic semiconductors: Ab initio spin-density functional theory. *Physical Review B* **79**, 045209 (2009).

4. Brinker, S., dos Santos Dias, M. & Lounis, S. The chiral biquadratic pair interaction. *New Journal of Physics* **21**, 083015 (2019).

5. Li, J. *et al.* Topological superconductivity induced by ferromagnetic metal chains. *Physical Review B* **90**, 235433 (2014).

6. Kim, H. *et al.* Toward tailoring majorana bound states in artificially constructed magnetic atom chains on elemental superconductors. *Sci Adv* **4**, eaar5251 (2018).
